# A clinical risk score of myocardial fibrosis predicts adverse outcomes in aortic stenosis

**DOI:** 10.1093/eurheartj/ehv525

**Published:** 2015-10-21

**Authors:** Calvin W.L. Chin, David Messika-Zeitoun, Anoop S.V. Shah, Guillaume Lefevre, Sophie Bailleul, Emily N.W. Yeung, Maria Koo, Saeed Mirsadraee, Tiffany Mathieu, Scott I. Semple, Nicholas L. Mills, Alec Vahanian, David E. Newby, Marc R. Dweck

**Affiliations:** 1 British Heart Foundation/University Centre for Cardiovascular Science, University of Edinburgh, Chancellor's Building, 49 Little France Crescent, Edinburgh EH16 4SB, UK; 2 National Heart Center Singapore, Singapore, Singapore; 3 Cardiology Department, AP-HP, Bichat Hospital, Paris, France; 4 Biochemistry Department, AP-HP, Tenon Hospital, Paris, France

**Keywords:** Aortic stenosis, Midwall myocardial fibrosis, Cardiovascular magnetic resonance imaging, High-sensitivity troponin I concentrations, Electrocardiogram strain

## Abstract

**Aims:**

Midwall myocardial fibrosis on cardiovascular magnetic resonance (CMR) is a marker of early ventricular decompensation and adverse outcomes in aortic stenosis (AS). We aimed to develop and validate a novel clinical score using variables associated with midwall fibrosis.

**Methods and results:**

One hundred forty-seven patients (peak aortic velocity (*V*_max_) 3.9 [3.2,4.4] m/s) underwent CMR to determine midwall fibrosis (CMR cohort). Routine clinical variables that demonstrated significant association with midwall fibrosis were included in a multivariate logistic score. We validated the prognostic value of the score in two separate outcome cohorts of asymptomatic patients (internal: *n* = 127, follow-up 10.3 [5.7,11.2] years; external: *n* = 289, follow-up 2.6 [1.6,4.5] years). Primary outcome was a composite of AS-related events (cardiovascular death, heart failure, and new angina, dyspnoea, or syncope). The final score consisted of age, sex, *V*_max_, high-sensitivity troponin I concentration, and electrocardiographic strain pattern [*c*-statistic 0.85 (95% confidence interval 0.78–0.91), *P* < 0.001; Hosmer–Lemeshow *χ*^2^ = 7.33, *P* = 0.50]. Patients in the outcome cohorts were classified according to the sensitivity and specificity of this score (both at 98%): low risk (probability score <7%), intermediate risk (7–57%), and high risk (>57%). In the internal outcome cohort, AS-related event rates were >10-fold higher in high-risk patients compared with those at low risk (23.9 vs. 2.1 events/100 patient-years, respectively; log rank *P* < 0.001). Similar findings were observed in the external outcome cohort (31.6 vs. 4.6 events/100 patient-years, respectively; log rank *P* < 0.001).

**Conclusion:**

We propose a clinical score that predicts adverse outcomes in asymptomatic AS patients and potentially identifies high-risk patients who may benefit from early valve replacement.

**See page 724 for the editorial comment on this article (doi:10.1093/eurheartj/ehv578)**

## Introduction

In response to aortic stenosis (AS), left ventricular (LV) hypertrophy initially occurs as a compensatory response to maintain wall stress and cardiac output. Ultimately, the LV decompensates and heart failure ensues. The transition from adaptive LV hypertrophy to heart failure is characterized by myocyte death and myocardial fibrosis^1–3^ and is an important determinant of symptoms and adverse clinical outcomes. Myocardial fibrosis can be detected non-invasively using cardiovascular magnetic resonance (CMR), and increasing evidence has demonstrated the presence of midwall fibrosis as an early marker of ventricular decompensation and predictor of adverse cardiovascular outcomes in patients with AS.^4–9^

Despite its potential prognostic value, the widespread clinical utility of CMR is sometimes limited by cost, availability, and patient suitability. We have recently demonstrated two alternative and more widely available markers of LV decompensation that are closely associated with the presence of midwall fibrosis.^10,11^ In separate studies, high-sensitivity plasma cardiac troponin I (cTnI) concentrations and the presence of LV hypertrophy with strain pattern on the electrocardiogram (ECG strain) were both independently associated with midwall fibrosis on CMR and adverse cardiovascular events, over and above conventional prognostic markers in AS.^10,11^ While high-sensitivity cTnI was a sensitive marker (100%) of midwall myocardial fibrosis, the ECG strain pattern was very specific (99%). The integration of these objective markers of LV decompensation into a clinical predictive score therefore represents a potentially attractive strategy of risk stratifying asymptomatic patients with AS and guiding the optimal timing of aortic valve replacement (AVR).

Using a novel approach, we aimed to develop a predictive score comprising variables associated with midwall myocardial fibrosis on CMR: a pathophysiologically relevant marker of early decompensation and adverse outcomes in AS. We then validated the prognostic impact of this clinical score in two large independent cohorts of asymptomatic patients with AS.

## Methods

### Patient populations

Three cohorts of patients were used in the study. A cohort of patients undergoing CMR was used to develop the clinical score to determine the probability of midwall myocardial fibrosis (CMR derivation cohort). This score was based on simple and widely available cardiac investigations. The prognostic value of this clinical score was then validated in two independent outcome cohorts of asymptomatic patients with an ejection fraction of >50%: an internal outcome cohort from the south-east of Scotland and an external outcome cohort from the Bichat Hospital, Paris. The study was conducted in accordance with the Declaration of Helsinki and was approved by the local research ethics committee. Written informed consent was obtained in all patients.

#### Cardiovascular magnetic resonance derivation cohort

The CMR derivation cohort consisted of stable patients with mild-to-severe AS. Patients were recruited from the outpatient clinics at the Edinburgh Heart Centre from March 2012 (clinicalTrials.gov identifier NCT01755936). Patients who had other significant valvular heart disease (≥ moderate), contraindications to CMR or cardiomyopathies (acquired or inherited) were excluded. As this study aimed to identify variables of midwall myocardial fibrosis due to AS, we excluded patients with previous myocardial infarction based on clinical history and confirmed on CMR. Blood samples were taken at the time of CMR and clinical assessment.

#### Internal outcome cohort

This internal outcome cohort consisted of patients with AS initially recruited into the Scottish Aortic Stenosis and Lipid Lowering Trial, Impact of REgression study. In brief, 155 asymptomatic patients were recruited between March 2001 and April 2002 to investigate the effects of intensive lipid-lowering therapy on AS progression.^12^

#### External outcome cohort

The external outcome cohort comprised of asymptomatic patients with at least mild AS from the COFRASA and GENERAC studies (clinicalTrials.gov numbers NCT00338676 and NCT00647088, respectively). These patients were prospectively recruited since November 2006. Exclusion criteria were AS due to rheumatic valvular disease or radiotherapy, previous infective endocarditis, other significant valvular diseases (≥moderate), and severe respiratory or renal insufficiency.

### Electrocardiography

A standard 12-lead ECG was performed in all patients. Electrocardiogram strain was diagnosed with the Romhilt-Estes point system (≥5 points)^13^ and the presence of ≥1 mm concave downsloping ST depression with asymmetrical T-wave inversion in the lateral leads.^14^

### Echocardiography

All patients underwent comprehensive echocardiography to determine AS severity. Peak aortic jet velocity (*V*_max_) and the mean pressure gradient were determined by velocity time integral spectral Doppler, and the aortic valve area estimated using the continuity equation. The severity was assessed and classified according to the European Association of Echocardiography/American Society of Echocardiography guidelines.^15^

### High-sensitivity cardiac troponin I and natriuretic peptide assays

Plasma cTnI concentrations were determined across the three cohorts using a high-sensitivity assay (ARCHITECT_STAT_, Abbott Laboratories, IL, USA). The lower limit of detection for this assay was 1.2 ng/L and the concentration at 10% inter-assay imprecision was 4.7 ng/L.^16^ Concentrations lower than the detection levels were assigned a value of 1.2 ng/L. In the CMR derivation cohort, plasma brain natriuretic peptide (BNP) concentrations were determined using the Triage BNP assay (Biosite Inc., San Diego, CA, USA).^17^ In the internal and external outcome cohorts, plasma *N*-terminal pro-BNP concentrations were measured using the Elecsys 2010 analyzer (Roche Diagnostics Ltd, Lewes, UK).^18^ For both BNP assays, concentrations lower than the manufacturer-reported lower limit of detection were assigned the lowest value (5 pg/mL).

### Cardiovascular magnetic resonance in the cardiovascular magnetic resonance derivation cohort

Cardiovascular magnetic resonance was performed using a 3T scanner (MAGNETOM Verio, Siemens AG, Healthcare Sector, Germany). Short-axis cines from the mitral valve annulus to the apex were used to assess LV volume, function, and mass (balanced steady-state free precision sequence; 8 mm parallel slices with 2 mm gap). All measurements were indexed to body surface area (Argus Ventricular Function, Siemens AG Healthcare Sector, Erlangen, Germany). Cardiovascular magnetic resonance LV longitudinal function was assessed using a method previously described.^11^

The assessment of focal midwall myocardial fibrosis was performed using late gadolinium enhancement (LGE), 15 min following 0.1 mmol/kg of gadobutrol (Gadovist/Gadavist, Bayer Pharma AG, Germany). Two approaches were used: an inversion recovery fast gradient-echo sequence and a phase-sensitive inversion recovery sequence, performed in two phase-encoding directions to differentiate true late enhancement from artefact. The inversion time was optimized to achieve satisfactory nulling of the myocardium for the inversion-recovery images. Midwall LGE was determined qualitatively by two independent and experienced operators (C.W.L.C. and M.R.D.).

### Clinical outcomes

The primary outcome of the study was AS-related events: a composite of cardiovascular mortality, congestive heart failure, and new symptoms of angina, syncope, or dyspnoea. The secondary outcomes were all-cause mortality and cardiovascular mortality. All events in the internal outcome cohort were adjudicated from the General Register of Scotland and verified by two independent investigators. Any discrepancy was resolved by consensus. In the external outcome cohort, events were adjudicated by experienced cardiologists blinded to any biological or ECG information. Patients in the internal and external outcome cohorts were followed until September 2012 and December 2014, respectively and events were censored at the time of last patient contact or at the time of AVR.

### Statistical analysis

Baseline characteristics were reported as percentages for categorical variables, mean ± standard deviation or median (inter-quartile range) for continuous variables as appropriate. The distribution of all continuous variables was tested for normality using the Shapiro–Wilk test. Statistical analyses were performed using SPSS version 20 (IBM Corp., Armonk, NY, USA) and GraphPad Prism version 5.0 (GraphPad Software, San Diego, CA, USA). Statistical significance was taken as a two-sided *P* < 0.05.

#### Establishing determinants of midwall myocardial fibrosis

In the CMR derivation cohort, clinically relevant variables that demonstrated univariate association with midwall myocardial fibrosis (*P* < 0.20) were selected in the multivariate logistic model. Subsequently, backward elimination method was used to establish a best-fitting parsimonious model, providing the basis for the score. The diagnostic performance of the clinical score was assessed using the *c* statistic for discrimination (area under the receiver operating curve) and the Hosmer–Lemeshow goodness of fit for calibration. We then identified score thresholds at 98% sensitivity and 98% specificity for midwall myocardial fibrosis, accepting a combined false-positive and -negative rate of <5%. These values would define the risk categories of patients in the outcome cohorts.

#### Validation of clinical score and cardiovascular outcomes

**Figure 1:**
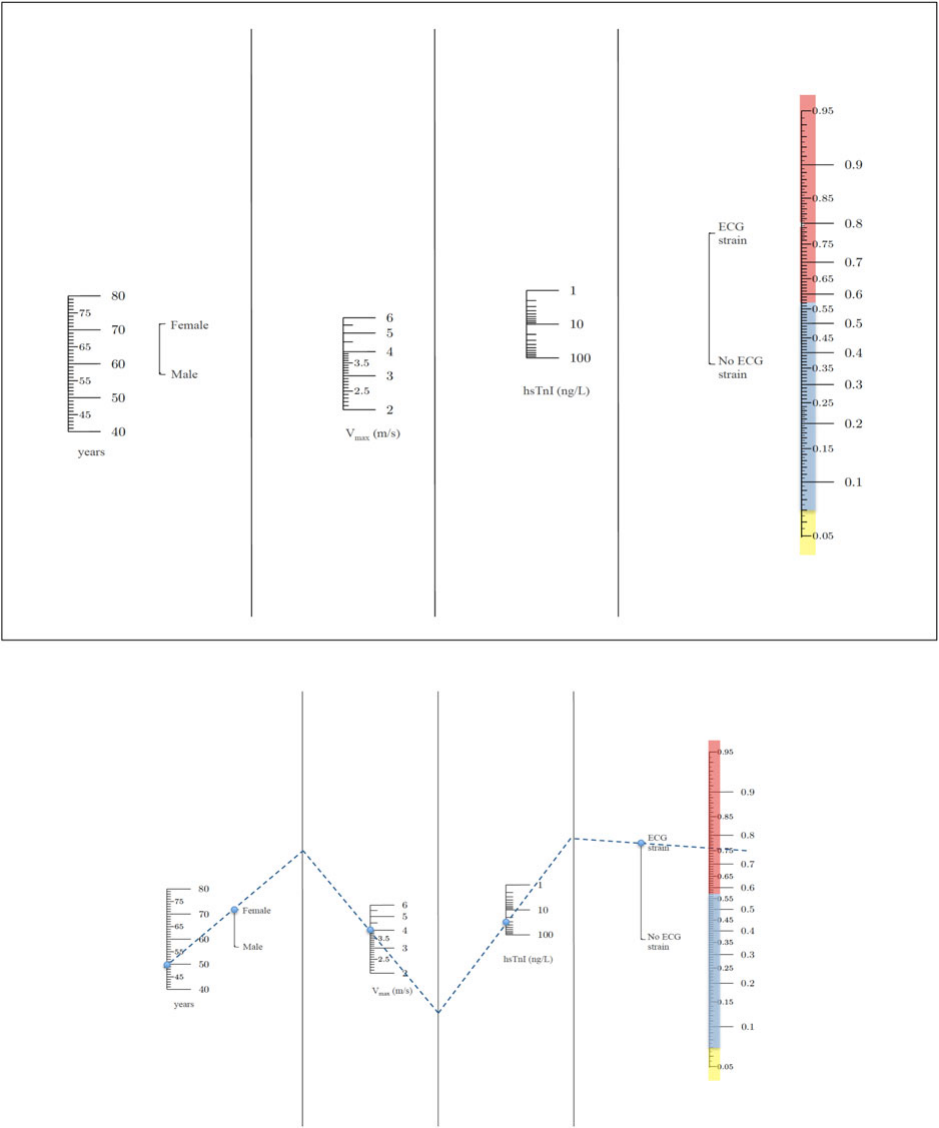
Nomogram for aortic stenosis clinical score. The risk probability can be also calculated using a nomogram. For example, a 50-year-old female patient with peak aortic jet velocity of 4.0 m/s, high-sensitivity cardiac troponin concentration of 30 ng/L and electrocardiogram strain pattern would have a risk probability of 78% (high risk).

Using our clinical score, the predicted probability (*P*) for midwall myocardial fibrosis was calculated for each patient in the internal and external outcome cohorts, according to the equation: $P=\exp^{y}/[1+\exp^{y}],$ where $y=\beta_{0}+\sum \beta_{i}X_{i},$ where *β*_0_ is the constant of the logistic equation, *β*_i_ is the regression coefficient of each variable, and *X*_i_ is the clinical model. In practice, the clinical score and the corresponding risk category for each patient were obtained easily using our online calculator (see Aortic Stenosis Risk Calculator, Supplementary material online) or a nomogram (*Figure 1*). The clinical score is also available in the mobile app Calculate by QxMD on iOS, Android and Windows (http://qx.md/calculate) and on the web at qxmd.com/as-risk-score. 

Patients with scores less than the threshold at 98% sensitivity for midwall myocardial fibrosis were classified as low risk. Conversely, patients with scores greater than the threshold at 98% specificity for midwall myocardial fibrosis were classified as high risk. All others were at intermediate risk. Time-to-first event survival curves associated with the different risk categories were estimated using the Kaplan–Meier method and compared with the log-rank test.

## Results

### Cardiovascular magnetic resonance derivation cohort

One hundred and sixty-six patients with AS were recruited in the CMR derivation cohort. We excluded 15 patients with myocardial infarction and 5 patients without blood samples (1 patient with myocardial infarction did not have blood samples). Compared with patients without midwall fibrosis (*n* = 103), those with midwall fibrosis (*n* = 44) had more severe AS and elevated markers of LV decompensation (*P* < 0.001 for all; *Table 1*). Thirty-seven patients in the CMR derivation cohort had symptoms consistent with severe AS. 

**Table 1:** Baseline characteristics of patients in the cardiovascular magnetic resonance cohort

	All patients (*N* = 147)	No midwall myocardial fibrosis (*N* = 103)	Midwall myocardial fibrosis (*N* = 44)	*P*
Clinical characteristics				
Age (years)	70 [63,76]	70 [63,76]	71 [65,78]	0.42
Male, *n* (%)	99 (68)	66 (67)	33 (70)	0.20
Diabetes mellitus, *n* (%)	21 (14)	15 (15)	6 (13)	0.64
CAD, *n* (%)	47 (32)	29 (29)	18 (38)	0.34
SBP (mmHg)	151 ± 21	151 ± 22	153 ± 19	0.41
hsTnI concentration (ng/L)	6.0 [3.6,11.6]	4.6 [3.2,8.0]	10.8 [6.6,26.5]	<0.001
BNP concentration (pg/mL)	24.7 [10.4,53.1]	21.8 [7.5,43.4]	34.4 [12.4,87.5]	0.01
ECG strain, *n* (%)	22 (15)	0	22 (46)	<0.001
Echocardiogrphy				
*V*_max_ (m/s)	3.9 [3.2,4.4]	3.7 [2.9,4.2]	4.1 [3.8,4.6]	<0.001
MPG (mmHg)	33 [22,43]	29 [17,40]	37 [29,50]	<0.001
AVA (cm^2^)	0.88 [0.73,1.11]	0.96 [0.74,1.20]	0.81 [0.73,0.91]	0.008
LVM_i_ (g/m^2^)	122 ± 32	116 ± 29	137 ± 34	<0.001
Diastolic function (*E*/*e*′)	12.6 [10.1,16.7]	11.7 [8.9,15.2]	14.5 [12.3,19.9]	<0.001
CMR				
EDV_i_ (mL/m^2^)	69 [61,78]	68 [60,76]	72 [65,88]	0.03
ESV_i_ (mL/m^2^)	23 [18,27]	22 [18,26]	24 [20,30]	0.08
SV_i_ (mL/m^2^)	47 [41,54]	46 [40,53]	49 [43,58]	0.05
LVEF (%)	67 [63,71]	68 [64,71]	67 [63,71]	0.55
Longitudinal function (mm)	12.3 ± 2.9	13.0 ± 2.6	10.9 ± 3.1	<0.001
LVM_i_ (g/m^2^)	87 [73,99]	80 [67,91]	101 [93,118]	<0.001
LVM/EDV (g/mL)	1.26 ± 0.27	1.18 ± 0.24	1.42 ± 0.27	<0.001

CAD, coronary artery disease; SBP, systolic blood pressure; hsTnI, high-sensitivity cardiac troponin I; BNP, brain natriuretic peptide; NT-proBNP, N-terminal proBNP; ECG strain, electrocardiographic left ventricular hypertrophy with strain; *V*_max_, peak aortic jet velocity; MPG, mean pressure gradient; AVA, aortic valve area; LVM_i_, indexed left ventricular mass; EDV_i_, indexed end-diastolic volume; ESV_i_, indexed end-systolic volume; SV_i_, indexed stroke volume; LVEF, left ventricular ejection fraction.

The final clinical score of age, sex, high-sensitivity cTnI concentrations (log_10_ transformed), *V*_max_ (log_e_ transformed), and ECG strain demonstrated excellent discrimination (*c* statistics = 0.85; 95% confidence interval 0.78–0.91; *P* < 0.001) and calibration (Hosmer–Lemeshow *χ*^2^ = 7.33; *P* = 0.50; *Table 2*), and it outperformed other determinants of midwall myocardial fibrosis (*Table 3*). The risk probabilities that corresponded to 98% sensitivity and 98% specificity for midwall fibrosis were 7.0 and 57.0%, respectively. On this basis, 14% of patients (*n* = 21) in the CMR derivation cohort were at low risk of midwall myocardial fibrosis (risk score < 7.0%) and 19% (*n* = 28) at high risk (risk score >57.0%). Among those at intermediate risk (*n* = 98), 18% had midwall myocardial fibrosis on CMR. Of note, the clinical score correlated well with diastolic function (*r* = 0.31; *P* < 0.001), CMR longitudinal function (*r* = −0.42; *P* < 0.001), and fibrosis volume assessed using myocardial T1 mapping (*r* = 0.66; see Supplementary material online). 

**Table 2:** Clinical determinants of midwall myocardial fibrosis

Variable	Univariate		Clinical model^a^	
	Regression coefficient (standard error)	*P*	Regression coefficient (standard error)	*P*
Age (years)	0.021 (0.016)	0.19	0.047 (0.027)	0.08
Male	0.692 (0.400)	0.08	1.356 (0.651)	0.04
SBP (mmHg)	0.007 (0.008)	0.64	–	–
Presence of CAD	0.412 (0.370)	0.27	–	–
*V*_max_ (m/s)^b^	3.514 (0.922)	<0.001	2.319 (1.282)	0.07
hsTnI concentration (ng/L)^c^	2.133 (0.486)	<0.001	0.935 (0.604)	0.12
BNP concentration (pg/mL)^b^	1.056 (0.424)	0.01	–	–
ECG strain	4.364 (1.046)	<0.001	3.616 (1.145)	0.002
Constant	–	–	−9.387 (2.801)	0.001

For abbreviations, see *Table 1*.

^a^Brain natriuretic peptide was selected in the initial multivariate model; but it was not retained in the final clinical score using backward elimination.

^b^Values were log_e_ transformed.

^c^Values were log_10_ transformed.

**Table 3:** Performance of determinants associated with midwall myocardial fibrosis

	Discrimination		Calibration Hosmer–Lemeshow goodness-of-fit test	
	*c* statistics (95% CI)	*P*	*χ* ^2^	*P*
*V*_max_	0.70 (0.62–0.79)	<0.001	6.5	0.58
BNP concentration	0.63 (0.52–0.74)	0.016	13.5	0.06
hsTnI concentration	0.76 (0.68–0.85)	<0.001	15.0	0.06
ECG strain	0.71 (0.62–0.81)	<0.001	NA	NA
Clinical score^a^	0.85 (0.78–0.91)	<0.001*	7.3	0.50

For abbreviations, see *Table 1*.

^a^The clinical score consisted of age, sex, *V*_max_, hsTnI concentrations, and ECG strain.

*P < 0.05 when compared with *V*_max_, BNP concentration and ECG strain; *P* = 0.07 when compared with hsTnI concentration.

### Association between clinical score and adverse events

#### Internal outcome cohort

In this cohort, 127 asymptomatic patients were analysed (69 [62,75] years, 70% males, *V*_max_ 3.4 [2.9,4.0] m/s) after excluding patients without blood samples (*n* = 24). Using the two risk thresholds established from the CMR derivation cohort, 13% of the patients (*n* = 17) in the internal outcome cohort were classified as low risk and 15% (*n* = 19) as high risk. While no low-risk patients had *V*_max_ ≥4.0 m/s, 42% of high-risk patients had *V*_max_ between 3.0 and 3.9 m/s (*Table 4*). 

**Table 4:** Relevant characteristics of patients in the cardiovascular magnetic resonance and outcome cohorts risk stratified by probabilities of midwall fibrosis

Low risk (probability <7%)	CMR cohort (*N* = 21)	Internal outcome cohort (*N* = 17)	External outcome cohort (*N* = 45)
Age (years)	63 [48,69]	57 [49,66]	70 [61,74]
Males, *n* (%)	3 (14)	3 (18)	4 (9)
*V*_max_ (m/s)	2.8 [2.5,3.2]	3.0 [2.7,3.4]	2.6 [2.4,2.9]
ECG strain, *n* (%)	0	0	0
hsTnI concentration (ng/L)	2.1 [1.5,4.0]	5.4 [4.0,6.6]	4.1 [3.0,6.4]
Patients with *V*_max_ 3.0–3.9 m/s, *n* (%)	5 (24)	10 (59)	6 (13)
Patients with *V*_max_ ≥4.0 m/s, *n* (%)	1 (5)	0	2 (4)
AS-related events, *n* (%)	NA	3 (18)	7 (16)
**Intermediate risk (probability 7–57%)**	**CMR cohort (*N* = 98)**	**Internal outcome cohort (*N* = 91)**	**External outcome cohort (*N* = 221)**
Age (years)	71 [66,77]	69 [63,75]	75 [67,80]
Males, *n* (%)	74 (76)	71 (78)	186 (84)
*V*_max_ (m/s)	3.8 [3.3,4.2]	3.3 [2.8,4.0]	3.1 [2.6,3.5]
ECG strain, *n* (%)	0	0	0
hsTnI concentration (ng/L)	5.3 [3.8,9.5]	7.6 [5.8,12.2]	7.0 [5.0,11.0]
Patients with *V*_max_ 3.0–3.9 m/s, *n* (%)	47 (48)	40 (44)	96 (43)
Patients with *V*_max_ ≥4.0 m/s, *n* (%)	37 (38)	24 (26)	27 (12)
AS-related events, *n* (%)	NA	47 (52)	56 (25)
**High risk (probability >57%)**	**CMR cohort (*N* = 28)**	**Internal outcome cohort (*N* = 19)**	**External outcome cohort (*N* = 23)**
Age (years)	71 [62,78]	75 [66,77]	79 [72,84]
Males, *n* (%)	22 (79)	15 (79)	19 (83)
*V*_max_ (m/s)	4.6 [4.1,5.1]	4.1 [3.5,4.4]	3.9 [3.1,5.4]
ECG strain, *n* (%)	22 (92)	19 (100)	17 (74)
hsTnI concentration (ng/L)	25.2 [10.1,46.7]	17.3 [10.5,29.6]	14.0 [9.0,21.0]
Patients with *V*_max_ 3.0–3.9 m/s, *n* (%)	4 (14)	8 (42)	7 (30)
Patients with *V*_max_ ≥4.0 m/s, *n* (%)	24 (86)	10 (53)	11 (48)
AS-related events, *n* (%)	NA	12 (63)	13 (57)

For abbreviations, see *Table 1*.

**Figure 2:**
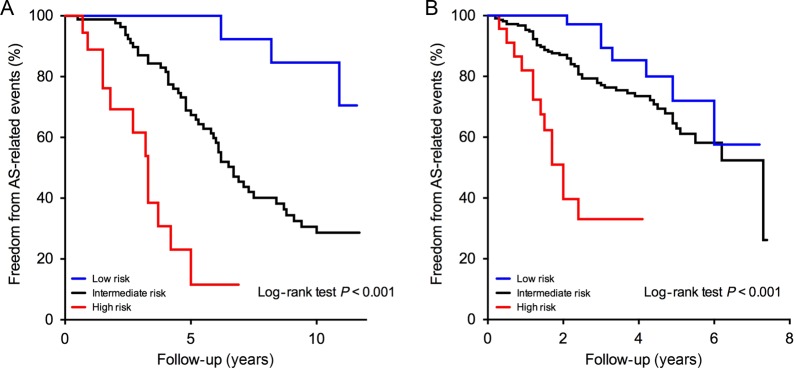
Aortic stenosis-related events stratified according to the risk of midwall myocardial fibrosis in the internal outcome cohort (*A*) and external outcome cohort (*B*).

There were 62 AS-related events (cardiovascular mortality, *n* = 26; congestive heart failure and new symptoms, *n* = 36) over 10.3 [5.7,11.2] years of follow-up (704.6 patient-years; 8.8 events/100 patient-years). In low-risk patients, only three AS-related events were observed. Conversely, high-risk patients had over a 10-fold increase in the AS-related event rate (23.9 vs. 2.1 events/100 patient-years in low-risk patients; log rank *P* < 0.001; *Figure 2*; *Table 4*), which all occurred early and within the first 5 years. 

**Figure 3:**
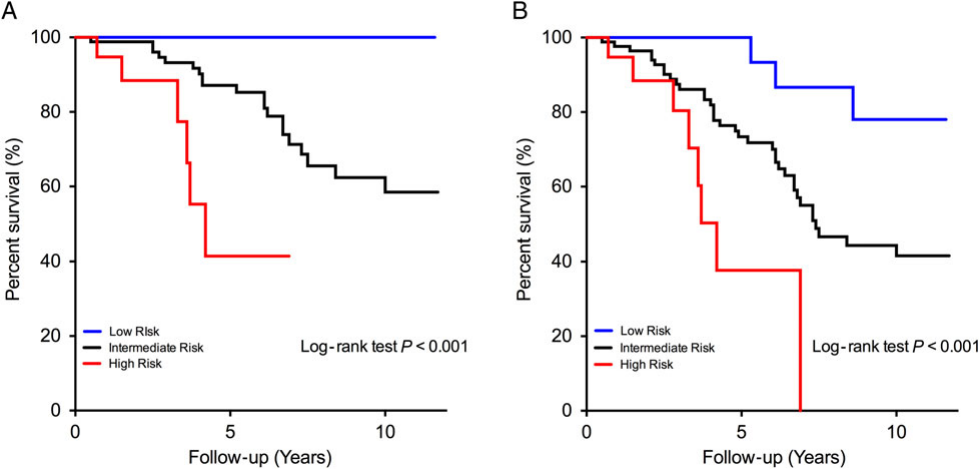
Cardiovascular (*A*) and all-cause mortality (*B*) in the internal outcome cohort.

Similar findings were observed with mortality rates. Forty-six patients died (26 from cardiovascular causes) during follow-up. In the low-risk group, there were no cardiovascular deaths during the entire period of follow-up. Three patients died from non-cardiac causes with no deaths within the first 5 years. By comparison, mortality rates were ∼7-fold higher in high-risk patients (13.0 vs. 2.1 all-cause deaths/100 patient-years in low-risk patients; log rank *P* < 0.001; *Figure 3*), and cardiovascular causes accounted for more than two-thirds of the deaths. 

#### External outcome cohort

A total of 289 patients were analysed (74 [67,80] years, 72% males, *V*_max_ 3.0 [2.5,3.6] m/s), after excluding patients without blood samples (*n* = 18) or interpretable ECGs (*n* = 25; *Table 5*). In this cohort, 16% (*n* = 45) and 8.0% (*n* = 23) of the patients were classified as low and high risk, respectively. Two low-risk patients had *V*_max_ = 4.0 m/s while 30% of high-risk patients had *V*_max_ between 3.0 and 3.9 (*Table 4*). Over 2.6 [1.6,4.5] years of follow-up (854.9 patient-years), there were 76 AS-related events (cardiovascular deaths, *n* = 9; congestive heart failure and new symptoms, *n* = 67) and an event rate similar to the internal outcome cohort (8.9 AS-related events/100 patient-years). The prognosis of low-risk patients was very favourable: only 7 events throughout the follow-up with no events in the first 2 years. Conversely, high-risk patients had substantially worse outcomes (31.6 vs. 4.6 AS-related events/100 patient-years in low-risk patients; log-rank test *P* < 0.001; *Figure 2*), and these events occurred very early (median time to event of 1.5 years). Compared with the internal outcome cohort, the external outcome cohort had a much shorter duration of follow-up and not unexpectedly, a considerably lower mortality rate that precluded further detailed analysis. 

**Table 5:** Baseline characteristics of patients in the internal and external outcome cohorts

	Internal outcome cohort (*N* = 127)	External outcome cohort (*N* = 289)	*P*
Clinical characteristics			
Age (years)	69 [62,75]	74 [67,80]	<0.001
Male, *n* (%)	89 (70)	209 (72)	0.88
Diabetes mellitus, *n* (%)	4 (3)	73 (25)	<0.001
CAD, *n* (%)	22 (17)	88 (30)	0.04
SBP (mmHg)	145 ± 19	126 ± 18	<0.001
hsTnI concentration (ng/L)	7.6 [5.7,13.4]	7.0 [4.8,11.0]	0.03
NT-proBNP concentration (pg/mL)	198 [121,531]	169 [73,419]	0.07
ECG strain, *n* (%)	19 (15)	18 (6)	0.06
Echocardiography			
*V*_max_ (m/s)	3.4 [2.9,4.0]	3.0 [2.6,3.6]	<0.001
Number of patients, *n* (%)			
<3.0 m/s	35 (27)	140 (48)	<0.001
3.0–3.9 m/s	58 (46)	109 (38)	
≥4 m/s	34 (27)	40 (14)	
MPG (mmHg)	24 [17,35]	21 [15,31]	0.01
AVA (cm^2^)	1.01 [0.72,1.28]	1.35 [1.10,1.60]	<0.001
LVM_i_ (g/m^2^)	142 [121,167]	116 [94,138]	<0.001
LVEF (%)	70 [64,78]	63 [63,68]	<0.01

For abbreviations, see *Table 1*.

### Incremental prognostic value of clinical score

**Figure 4:**
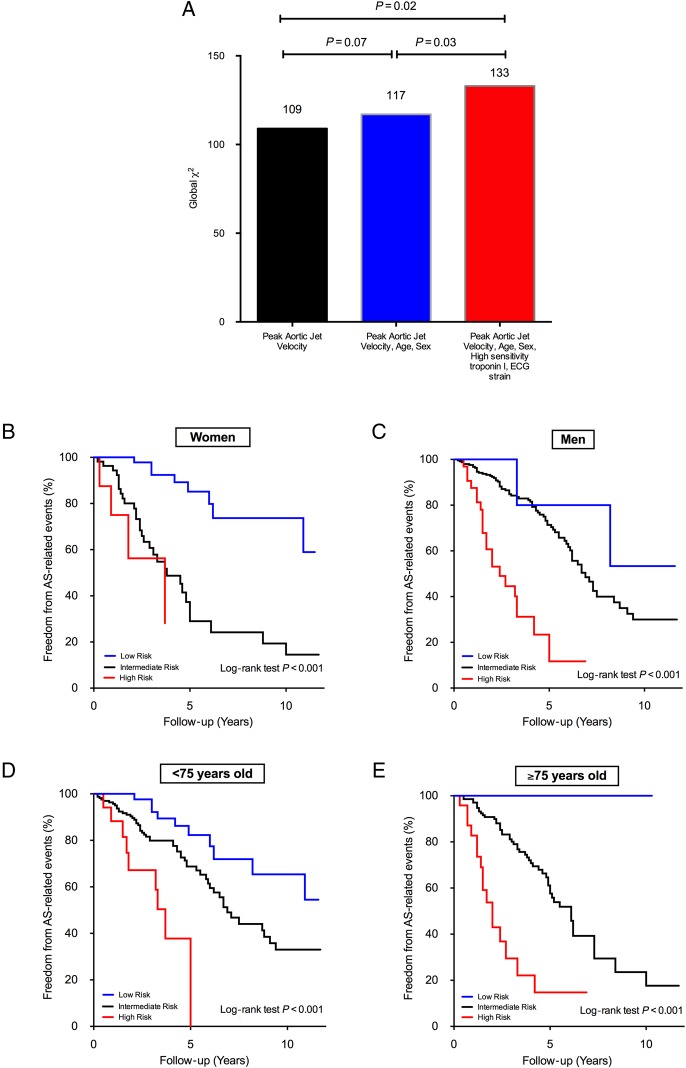
Incremental prognostic value of the clinical score. In the clinical score, high-sensitivity cardiac troponin I concentrations and electrocardiographic strain pattern provided incremental prognostic value over and above peak aortic jet velocity, age, and sex (*A*). While patients at low risk had very favourable prognosis, high-risk patients had very high event rate, regardless of sex (*B* and *C*) or median age (*D* and *E*).

We examined in greater detail the prognostic value of the clinical score. Addition of high-sensitivity cTnI and ECG strain in the score provided incremental prognostic value, over and above *V*_max_, age and sex (global *χ*^2^ increased from 117 to 133; *P* = 0.03; *Figure 3*). In particular, across the two outcome cohorts, similar improvement in risk stratification was observed in patients stratified by either median age or sex (log-rank test *P* < 0.001 for all analyses; *Figure 4*). 

**Figure 5:**
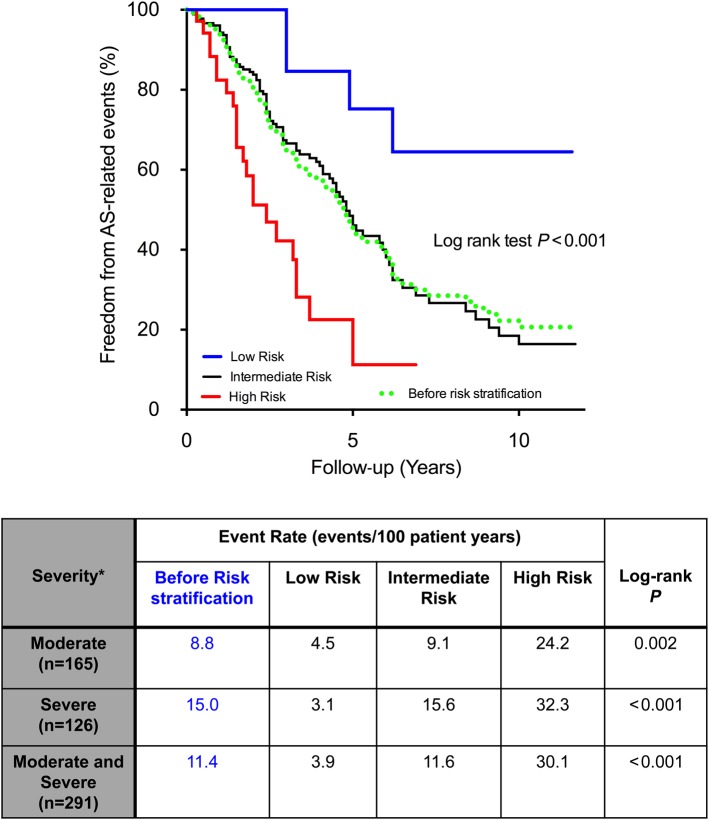
Improvement in risk stratification using the clinical score in patients with moderate and severe aortic stenosis. Compared with the natural history of patients with moderate/severe aortic stenosis (green dotted line), the clinical risk score demonstrated significant improvement in identifying patients at low and high risk for adverse events. Furthermore, patients at intermediate risk had an event rate very similar to that prior to risk stratification. This supported the incremental role of the clinical score over the traditional assessment of aortic stenosis severity.

Among patients with at least moderate AS (either *V*_max_ ≥3.0 m/s or aortic valve area ≤1.5 cm^2^), the clinical score further improved risk-stratification and identified patients with very low- and high-event rates (log rank *P* < 0.001 for both; *Figure 5*). Of note, the remaining patients at intermediate risk had an event rate almost identical to the natural history of the patients with moderate and severe AS (green dotted line; *Figure 5*). 

## Discussion

In a large CMR cohort of patients with AS, we have proposed the first clinical score consisting of variables associated with midwall myocardial fibrosis, an early and important marker of LV decompensation. This simple clinical score demonstrated excellent diagnostic performance for midwall myocardial fibrosis on CMR. Using the novel thresholds and across >400 asymptomatic patients (1560 patient-years), those at high risk (11% of patients in both outcome cohorts) had extremely poor outcomes while low-risk patients (16%) had very favourable prognosis. The clinical score has demonstrated important prognostic information in identifying patients who either may benefit from early AVR or can continue conservative surveillance.

Current guidelines recommend AVR in patients with severe AS and the evidence of LV decompensation based on either symptoms or a systolic ejection fraction <50%.^19,20^ However, symptoms are often difficult to elucidate in the elderly in whom adequate exercise stress testing may also be challenging. Furthermore, a low ejection fraction is a late manifestation and frequently irreversible. There is therefore considerable interest in examining novel and objective markers of LV decompensation to identify patients who may benefit from early AVR.^1,2^ The transition from hypertrophy to heart failure in AS is driven by progressive myocyte cell death and myocardial fibrosis. Cardiovascular magnetic resonance is able to visualize the latter directly, making it an attractive imaging modality to detect early decompensation. Indeed, we and others have reported that midwall fibrosis on CMR is not only associated with multiple features of LV decompensation but also an adverse prognosis in patients with AS.^4–9^

Unfortunately, the limited availability and relatively high costs of CMR may make routine surveillance impractical for all patients with AS. Consequently, a clinical score that is associated with midwall fibrosis is potentially attractive, particularly one that can also demonstrate prognostic value. In this study, we have developed such a score consisting of variables that can easily be obtained in routine clinical care. In addition to age, sex, and AS severity, both high-sensitivity cTnI and ECG strain pattern were retained in the final model as independent predictors of midwall fibrosis, consistent with recent literature.^10,11^ Rather than individual determinants, an integrated approach of using the clinical score performed best at identifying midwall myocardial fibrosis. In particular, one cannot simply rely on the traditional markers of AS severity (such as *V*_max_) as the magnitude of the hypertrophic response and the rate of LV decompensation are highly variable between patients.^1,21–23^ Although plasma BNP concentrations were associated with midwall myocardial fibrosis, the association was absent when other variables were considered. It is likely that BNP and NT-proBNP are released in the later stages of LV decompensation when symptoms develop and are therefore, not sensitive markers of midwall myocardial fibrosis or LV decompensation at an earlier state of the disease.

After the score was derived, we further established novel thresholds that might risk-stratify patients according to the probability of myocardial fibrosis. We have decided *a priori* to use stringent thresholds to define the high- and low-risk categories in order to minimize the false-positive and -negative rates of midwall fibrosis (<5%) and to maximize the score's ability to confidently identify low- and high-risk patients.

The prognostic ability of the clinical score and associated thresholds was then validated across two independent cohorts of >400 patients. To our knowledge, this is the largest validation cohort used to test a clinical score in AS. Low-risk patients identified by the score had a favourable prognosis: 16% of them had an AS-related event and only one cardiovascular death over a median time of 4.3 years. Conversely, high-risk patients had very poor outcomes: 67% of them had either an AS-related event or died over a median of 1.9 years, and these events occurred early. The clinical score demonstrated similar findings regardless of age and sex, and provided incremental prognostic information over conventional echocardiographic assessment of AS severity. Importantly, these improvements in risk stratification were observed in patients with moderate and severe disease.

### Clinical implications

Our observations have indirectly strengthened the prognostic association between CMR midwall fibrosis and cardiovascular outcomes. Potentially, asymptomatic patients with advanced AS can initially be risk stratified using this clinical score. Patients at low risk can be managed conservatively with regular reassessment of risk, while those at high risk (particularly those with severe AS) can be considered for early AVR. Finally, patients with intermediate-risk scores can undergo further risk stratification (such as CMR to definitively assess the presence of midwall myocardial fibrosis, computed tomography aortic valve calcium scores, or exercise stress testing). Our risk score will therefore guide clinical management in 25–30% of patients with AS, without the need for further investigations. This is a cost-effective strategy to guide the timing of AVR using more objective markers of LV decompensation. Ultimately, such an approach will need to be tested in a randomized controlled trial.

### Study limitations

This study is limited by a relatively short duration of follow-up in the external outcome cohort. Therefore, the lower mortality rates in the external outcome cohort precluded further detailed analysis. We had excluded patients with prior myocardial infarction from the CMR derivation cohort so as to derive an accurate clinical score of midwall myocardial fibrosis due to AS. Nevertheless, the findings remained unchanged when patients with prior myocardial infarction were included in the derivation of the score (see Supplementary material online). Finally, CMR was not performed in the two outcome cohorts and we were unable to reconfirm the presence of midwall fibrosis in these patients.

## Conclusions

We have developed a clinical risk score consisting of variables associated with midwall myocardial fibrosis. This score demonstrates strong prognostic information in asymptomatic patients with AS and holds major potential in identifying those who may benefit from early AVR.

## Supplementary material

Supplementary material is available at *European Heart Journal* online.

## Authors’ contribution

C.C., D.M.-Z., A.S., T.M.: performed statistical analysis. D.E.N., M.R.D.: handled funding and supervision. D.M.-Z., T.M., E.N.W.Y., M.K., G.L., S.B.: acquired the data. C.C., D.M.-Z.: conceived and designed the research. C.C., D.M.-Z., D.N., M.D.: drafted the manuscript. C.C., D.M.-Z., A.S., G.L., S.B., S.M., S.S, N.M., A.V., D.N., M.D.: made critical revision of the manuscript for key intellectual content. S.S.: optimize MRI sequences crucial for the study. S.M.: read the MRI images alongside Calvin Chin and Marc Dweck—the names of the authors who did anything else on the manuscript other than what we have listed.

## Funding

A.S.V.S., N.L.M., D.E.N., and M.R.D. are supported by the British Heart Foundation (CH/09/002, FS/10/024, FS/10/26, and FS/14/78/31020). D.E.N. holds a Wellcome Trust Senior Investigator Award (WT103782AIA). C.W.L.C. is supported by the National Research Foundation-Ministry of Health, Singapore. The Wellcome Trust Clinical Research Facility and the Clinical Research Imaging Centre are supported by the NHS Research Scotland through NHS Lothian. The COFRASA (clinicalTrials.gov number NCT 00338676) and GENERAC (clinicalTrials.gov number NCT00647088) studies are supported by grants from the Assistance Publique–Hôpitaux de Paris (PHRC National 2005 and 2010 and PHRC regional 2007). Funding to pay the Open Access publication charges for this article was provided by British Heart Foundation.

**Conflict of interest**: A.S.V.S., G.L., and N.L.M. received speaker fees from Abbott Laboratories, and N.L.M. has acted as a consultant for Beckman-Coulter.

## Supplementary Material

Supplementary Data
