# Supplementary material for: A clinical risk score of myocardial fibrosis predicts adverse outcomes in aortic stenosis
Source: Eur Heart J. 2015 Oct 21;37(8):713–23. doi: 10.1093/eurheartj/ehv525 (PMC4761400; doi:10.1093/eurheartj/ehv525)

**A CLINICAL RISK SCORE OF MYOCARDIAL FIBROSIS PREDICTS ADVERSE OUTCOMES IN AORTIC STENOSIS**

**ONLINE SUPPLEMENTAL DATA**

**Inclusion of Patients with Previous Myocardial Infarction in the Derivation of the Clinical Score**

There were 15 patients with previous myocardial infarction in the CMR Derivation Cohort. In a further analysis, we have included these patients in the derivation of the clinical score. The clinical score demonstrated similar diagnostic performance for the presence of mid-wall fibrosis (*c* statistic 0.83 [0.75 to 0.90]; P<0.001 and Hosmer-Lemeshow χ^2^ 3.37; P<0.001), and in predicting adverse events in the two outcome cohorts (**Figure**).

However, we would advise caution in interpreting these results because the number of patients with previous myocardial infarction is inadequate to assess the true effects (if any) on the clinical risk score.

**
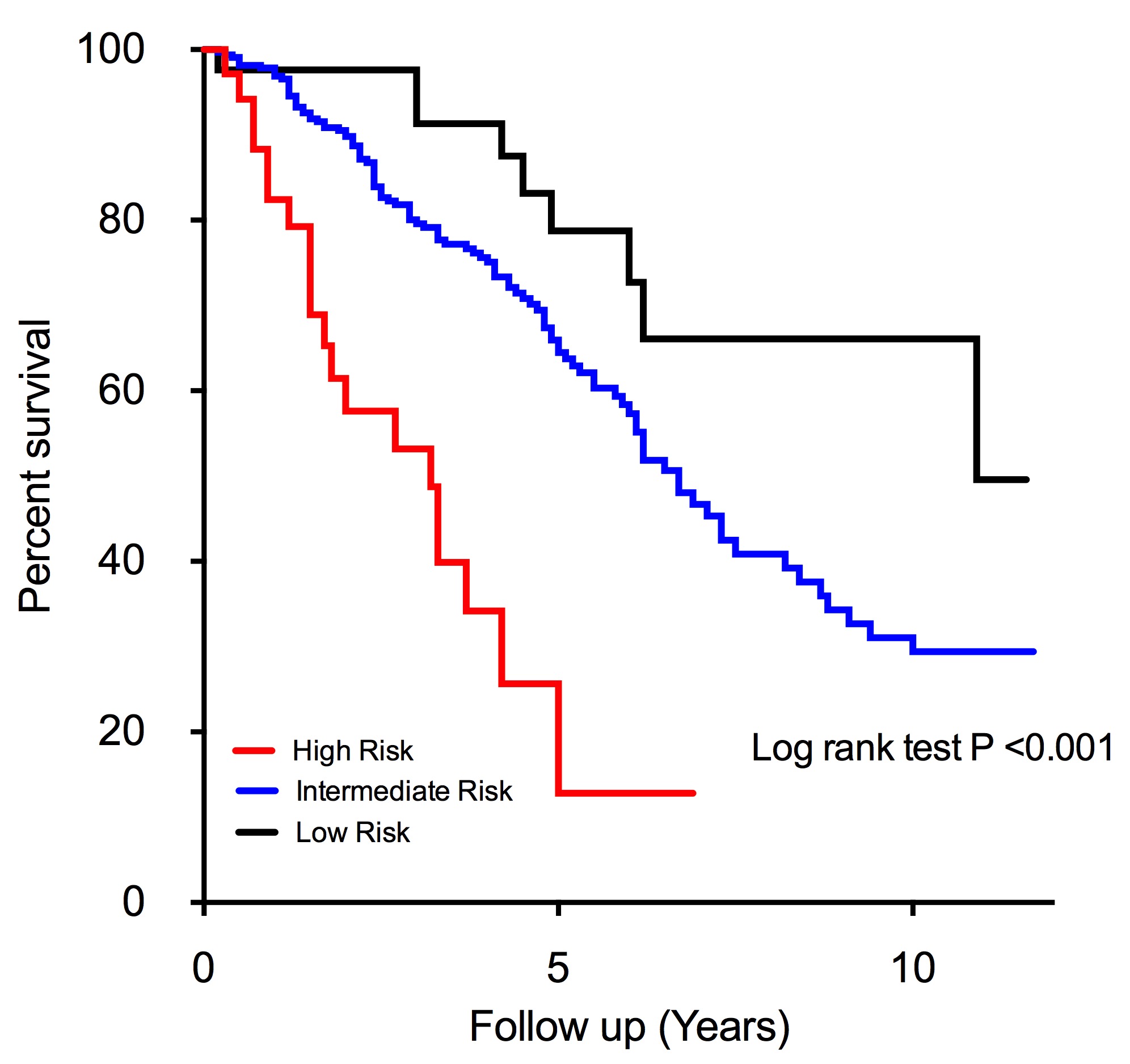
**

**Association Between the Clinical Score and Diffuse Myocardial Fibrosis Assessed Using Myocardial T1 Mapping**

Myocardial T1 mapping is a more sensitive cardiovascular magnetic resonance technique of assessing diffuse myocardial fibrosis. Patients in the CMR Derivation Cohort underwent myocardial T1 mapping in addition to late gadolinium enhancement according to the protocol previously published (Chin et al. Eur Heart J Cardiovasc Imaging. 2014;15:2339). In 16 patients, reassessment of risk using the clinical score and repeat cardiovascular magnetic resonance was performed after at least 1 year.

We calculated extracellular volume fraction (ECV) according to: ECV = partition coefficient x [1-hematocrit], where partition coefficient = [∆R1_myocardium_/∆R1_blood-pool_] and ∆R1 = (1/post-contrast T1-1/pre-contrast T1). Hematocrit was sampled at the time of cardiovascular magnetic resonance. Furthermore, fibrosis volume in each patient was derived using the following: ECV x left ventricular myocardial volume, where left ventricular myocardial volume = left ventricular mass /1.05 g/mL (Doltra et al. J Am Heart Assoc. 2014;3:e001353).

The clinical score has not only correlated positively with fibrosis volume assessed at baseline but also at subsequent follow-up (**Figure**). This data has provided additional support of the association between the clinical score and myocardial fibrosis.


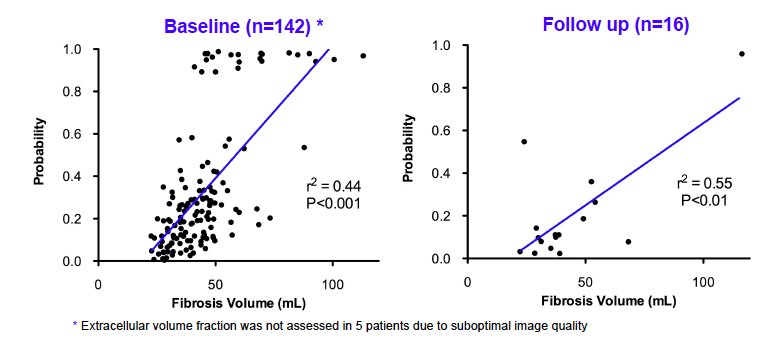

Supplement: Supplementary Data [file ehv525_supplementary_data.zip › ehv525supp.docx]
